# Giving samples or “getting checked”: measuring conflation of observational biospecimen research and clinical care in Latino communities

**DOI:** 10.1186/s12910-015-0041-9

**Published:** 2015-07-17

**Authors:** Sarah Knerr, Rachel M. Ceballos

**Affiliations:** Department of Health Services, 1959 NE Pacific St, Box 357660, Seattle, WA 98195 USA; Fred Hutchinson Cancer Research Center, 1100 Fairview Ave. N., M3-B232, PO Box 19024, Seattle, WA 98109 USA

**Keywords:** Hispanic/Latino, Diagnostic misconception, Psychometrics, Measure construction, Biospecimen donation

## Abstract

**Background:**

Expectations of receiving personal health information as a fringe benefit of biospecimen donation—termed diagnostic misconception—are increasingly documented. We developed an instrument measuring conflation of observational biospecimen-based research and clinical care for use with Latino communities, who may be particularly affected by diagnostic misconception due to limited health care access.

**Methods:**

The instrument was developed using prior qualitative research, revised through cognitive interviewing and expert review, and field tested in a convenience sample of 150 Latino adults in Eastern Washington State. It was further refined through exploratory factor analysis and validated against existing measures of genetic knowledge and researcher trust.

**Results:**

The final instrument demonstrated high internal consistency, evidence of content and construct validity, and no floor and ceiling effects. Individuals who were unemployed, spoke only Spanish, had no health insurance, received health care outside of traditional venues, and had good self-rated health received higher scores, indicating greater conflation of biospecimen-based research and clinical care.

**Conclusions:**

The ability to systematically measure beliefs related to diagnostic misconception will help facilitate ethically-informed efforts to recruit Latinos into biospecimen-based research studies.

**Electronic supplementary material:**

The online version of this article (doi:10.1186/s12910-015-0041-9) contains supplementary material, which is available to authorized users.

## Background

Observational studies relying on biological samples are increasingly used to better understand disease with a goal of improving population health. Though personalized results from biospecimen-based studies are not often clinically actionable and thus not made available to participants, prior research indicates that expectations of receiving meaningful health information as a fringe benefit of sample donation are not uncommon [1, 2]. Expectations of personal health benefit in the context of observational research have been termed diagnostic misconception [3], a variant of the therapeutic misconception that occurs in clinical trials when research participants either misunderstand or fail to appreciate key distinctions between the goals and guiding principles of research and clinical care [4, 5].

Our research group encountered a number of beliefs related to diagnostic misconception while conducting a qualitative study about observational biospecimen-based research participation with Latinos living on the United States (US)-Mexico border [6]. Our participants reported that they were extremely willing to provide biological samples for research, including blood, urine, stool, saliva, and buccal cells, but often equated providing a sample for research with undergoing a clinical evaluation. Sample donation was described as, ‘…a way of doing our check-ups to see if we’re in time to [detect] diseases’ ([6], p7). The conflation of research and clinical care influenced participants’ perceptions of the potential benefits of sample donation, which included receiving individualized information about medical diagnoses and future disease risk. Additionally, participants had trouble grasping the nature of observational research and were more familiar with clinical studies involving medical interventions.

Diagnostic misconception has important ethical dimensions, as inaccurate beliefs about the research process may unduly influence decisions to take part in research and impact the quality of the informed consent process [7]. Ethical concerns are magnified for Latino populations, who face substantial barriers to accessing health care in the US [8], but are increasingly sought after as research participants to improve generalizability, particularly in genomic studies [9, 10]. Factors influencing Latinos’ participation in studies involving the collection of biologic samples and accompanying phenotypic information have not been well studied [11, 12]. Whether expectations of receiving personally meaningful health information as a fringe benefit of participation drive sample donation in a coercive manner is particularly unclear.

In an effort to enable identification of beliefs related to diagnostic misconception in Latino communities, this study developed and validated a quantitative instrument measuring conflation of observational biospecimen-based research and clinical care. The availability of such an instrument will allow future research exploring the origins and consequences of diagnostic misconception and help facilitate ethically-informed recruitment efforts in medically underserved communities.

## Methods

### Conceptual model and item development

The conceptual model used to guide instrument development was informed by a review of the literature on biospecimen donation and diagnostic misconception, prior qualitative research conducted by our group on the US-Mexico border, and a quantitative measure of therapeutic misconception developed by Appelbaum et al. [13]. Common misconceptions about research with biological samples that were documented literature or observed in our prior work were grouped into three related domains. The first domain considers understanding of the distinctions between observational biospecimen-based research and clinical trials. The second and third domains are modeled on dimensions of the therapeutic misconception scale [13]. Specifically, the second domain concerns understanding of the purpose of biospecimen-based research, i.e. identifies the degree of conflation of the goals of biological sample collection for research (creating generalizable knowledge) and the goals of sample collection in clinical care (informing the care of the individual patient). The third domain concerns perceptions of the likelihood of receiving personal benefits in the form of individualized health information when providing a biospecimen for research.

We developed six items for the first domain and seven for the second and third. Items were written based on meaningful themes and quotations from our prior interviews and focus groups as well as relevant items developed for the therapeutic misconception scale [13]. Items were measured on 4-point Likert-like scales from strongly agree to strongly disagree. Based on our prior experience working with Latino communities in Eastern Washington State, we did not include ‘do not know’ as a response option because of respondents’ tendency to choose this option rather than make a potentially incorrect guess. Each item’s reading level was assessed in English using the Flesch-Kincaid grade level formula and kept as low as possible [14]. Items were reviewed for face-validity in English by two community-based participatory researchers who work extensively with Latino communities and three experts in measurement development. Items were then pre-tested in English (*n* = 5) and Spanish (*n* = 5) with members of the target population using standard cognitive interviewing techniques [15]. Interviews were conducted by a certified translator trained in cognitive interviewing, who also conducted all study translations.

### Field-testing

The instrument administered for field-testing included final versions of all items written for each domain along with single items assessing prior sample donation for research (‘yes’, ‘no’, or ‘don’t know’) and likelihood of providing a sample for research in the future (‘very likely’, ‘somewhat likely’, or ‘not likely’). We described biological samples as materials taken from the human body, including tissues like skin, hair, nails, or cheek cells and fluids like blood, urine, or salvia. Scientific research was described as a method of learning about health and how to prevent and treat diseases. The definitions were based on the National Cancer Institutes’ (NCI) Cancer 101 module on biospecimens and biobanking [16]. We also administered the Genetic Knowledge Index (GKI) [17] and Hall et al.’s scale measuring trust in medical researchers [18] along with standard demographic and health care access questions. The five-item version of the GKI has been previously used to assess basic knowledge about genetics in the general population [19]. Participants indicated whether five statements were true or false and a total score from 0 to 5 was calculated from their number of correct responses (Cronbach’s alpha = 0.56). The 4-item version of Hall et al.’s scale measuring trust in medical researchers, which has shown high reliability in national surveys, was modified for administration by removing the ‘do not know/can’t answer’ response option and changing ‘medical researcher’ to ‘scientists who do research with biological samples’ and ‘doctor’ to ‘scientist’. The items were measured on 4-point Likert-like scales from strongly disagree to strongly agree and summed, with higher overall scores indicating more trust in researchers (Cronbach’s alpha: 0.52).

We used a targeted recruitment strategy to obtain a sample of 150 self-identified Latino adults with an equivalent gender distribution to complete the survey instrument. Our sample size was chosen to provide adequate power for exploratory factor analyses (EFA) [20]. Recruitment efforts relied on an NCI Community Networks Program Center (CNPC) based in Sunnyside, Washington with strong connections to the Latino community there. Latinos living in this area of Eastern Washington State, called the lower Yakima Valley, are almost exclusively from Mexico and are similar to Latinos living on the border with respect to income, acculturation, education, and health care access [6, 11, 21]. Recruitment and survey administration were conducted by two CNPC staff members who are residents of the lower Yakima Valley and fluent in both English and Spanish. Study participants completed the surveys in person at community events, CNPC- sponsored health fairs, and local shopping facilities. Study materials were available in both English and Spanish and participants could use whatever language they felt most comfortable with. The survey was verbally administered, with participants also having their own copy to read if they desired, and took approximately 15-20 min to complete. This study was reviewed by the University of Washington Institutional Review Board who declared it exempt from ethics approval. Participants received a $15 gift-card as a thank your for their time. Completed surveys were periodically checked by the study team to ensure data quality and consistency in administration. When recruitment was completed the survey data was reviewed, coded, and entered into Stata 13 software [22]. A random sample of surveys (25 %) was reviewed to confirm the quality of entered data.

### Data analysis

Response distributions were examined for each item, with items coded so that higher scores indicated increasing misconceptions about research with biological samples. EFA was used to determine if items intended to measure the same domain were inter-correlated and to inform item selection for the final instrument [23]. We used the principal components factor method followed by Promax rotation, both implemented using the ‘factor’ command in Stata 13. [24, 25]. Promax rotation was chosen to be consistent with our conceptual model, which proposed that the three domains were interrelated [26].

In the initial factor extraction, six factors had eigenvalues above one and most items loaded onto the first factor. The remaining items were spilt so that those assessing accurate and inaccurate perceptions of biospecimen-based research loaded onto separate factors, suggesting that the observed factor structure may have been due to item wording and re-coding. The scree plot test had two changes in slope or “elbows”, one that occurred after the third factor and one that occurred after the sixth [27]. Based on these findings, we pursued two models moving forward. The first retained three factors aligning with the three domains originally proposed in our conceptual model. The second model retained one factor and reflected the overall degree of conflation of biospecimen research and clinical care. Rotated factor loadings from these models were used to create two reduced versions of the instrument (termed the 3-factor and the 1-factor solutions). For both models, items that had loadings above 0.60 on their own factor (and under 0.40 on any other factor for the 3-factor solution) were included in the final instrument. A second EFA was conducted on both reduced instruments and factor loading are reported for these analyses.

Item-to-scale correlations and Cronbach’s alpha statistics were calculated for both the 1- and 3-factor solutions. Additionally, mean subscores for each dimension in the 3-factor solution and their correlation with the total score were calculated along with Cronbach’s alpha statistics. To assess construct validity correlations with the GKI and Hall et al.’s scale measuring trust in medical researchers were examined. In our qualitative work on the US-Mexico border individuals who knew the least about research were often the most trusting of researchers and willing to provide biospecimens. Thus, we hypothesized that increasing misconceptions about biospecimen-based research as measured by the 3-factor solution would be negatively correlated with genetic knowledge and positively correlated with researcher trust. Subscores for all three domains would follow the same patterns, with the exception of lay understanding of research, which would be uncorrelated with researcher trust. Similarly, we hypothesized that increasing conflation of research with biological samples and clinical care as measured by the 1-factor solution would be negatively correlated with genetic knowledge and positively correlated with researcher trust.

## Results

### Participants

Descriptive characteristics for the cognitive interview and survey participants are shown in Table 1. Participants reflected the demographic profile of Latinos living in the lower Yakima Valley, with most having a high school education or less, an annual household income under $35,000, and either government health insurance (Medicare, Medicaid, or coupons) or no health insurance.

**Table 1:** Cognitive interview and survey participant characteristics

		Interview	Survey
		N	N, %
Overall		10	150
Age (Mean, SD)		31.6, 10	38.8, 15
Gender	Female	7	75, 50
Employment	Full-time	8	68, 45
	Part-time	0	30, 20
	Unemployed	2	52, 35
Years education	≤4	1	20, 13
	5-8	1	39, 26
	9-12	2	75, 50
	≥13	6	16, 11
Annual household income	<$15,000	0	63, 42
	$15,000 -$34,999	4	71, 47
	$35,000 -$49,999	2	13, 9
	>$50,000	4	3, 2
Primary language spoken	Only Spanish	2	38, 25
	Spanish better than English	1	36, 24
	Both	3	39, 26
	English better than Spanish	4	32, 21
	Only English	0	5, 3
Health insurance	Private	7	30, 20
	Government	2	77, 51
	Both	0	5, 3
	None	1	38, 25
Usual source of care	Doctor’s office	4	41, 27
	Clinic	6	97, 65
	Hospital	0	6, 4
	Other^*a*^	0	6, 4
Has regular doctor	Yes	5	112, 75
Self-rated health	Excellent or very good	2	51, 34
	Good	2	65, 43
	Fair or poor	5	34, 23
	Don’t know	1	0, 0
Provided a sample for research in the past	Yes	4	16, 11
	No	4	129, 86
	Don’t know	2	5, 3
Would provide a sample for research in the future	Very likely	7	58, 39
	Somewhat likely	3	72, 48
	Not likely	0	20, 13

Numbers may not sum to 100 due to rounding error

^*a*^Other: Pharmacy, traditional medicine/sobadores, family/friends, or prefer not to answer

### Instrument psychometrics

The 20 piloted items are presented by domain in Additional file 1: Table S1-S3 included in Additional file 1 along with item-level response data. Missing responses were rare and always occurred at the end of each domain. Responses in all domains tended to be skewed towards ‘strongly agree’ and ‘agree’, regardless of whether the item assessed accurate or inaccurate perceptions of biospecimen research. More than half the respondents ‘agreed’ or ‘strongly agreed’ with 67 % of the lay understanding items, 86 % if the purpose items, and 100 % of the benefits items.

Two of the items were highly skewed and excluded from the EFA. The Kaiser-Meyer-Olkin (KMO) statistic for the remaining 18 items was 0.77 and Barlett’s test of sphericity rejected the null hypothesis, indicating underlying data structure sufficient for EFA [26]. Table 2 provides factor loadings and correlations with domain scores and total scores for the 8 items retained in the 3-factor solution. The first factor (benefits) accounted for 33.9 % of item variance, the second factor (purpose) accounted for 20.3 %, and the third (lay understanding) accounted for 18.3 %. Items included in the 3-factor solution had high loadings on their own factor and high correlations with domain scores. But, correlations with the total score and Cronbach’s alphas were low for the lay understanding and purpose domains. Alpha statistics were 0.550 for lay understanding, 0.511 for purpose, 0.808 for benefits, and 0.589 for the overall 8-item scale. Table 3 provides factor loadings and correlations with total scores for the 6 items retained in the 1-factor solution. The first factor accounted for 56.7 % of item variance and Cronbach’s alpha statistic for the 6-item scale was 0.844.

**Table 2:** Final scale characteristics for three-factor solution

Item	Mean (SD)	Factor loadings			Correlation with domain score^*d*^	Correlation with total score^*d,e*^
		B^*a*^	LU^*b*^	P^*c*^		
1. A person must go to the hospital to give a biological sample for scientific research.^*f*^	2.10 (0.73)		0.710		0.826	0.614
2. A person must be invited by a doctor to give a biological sample for scientific research.^*f*^	2.50 (0.79)		0.860		0.837	0.464
3. Scientific research using biological samples is done to learn about what causes disease, not about each person who gives a biological sample.	1.89 (0.64)			0.863	0.855	0.047
4. A scientific researcher’s number one goal is to learn more about how to fight disease.	1.63 (0.54)			0.743	0.784	0.145
5. Researchers will always tell people if their biological sample shows risk for disease.^*f*^	3.01 (0.71)	0.699			0.718	0.561
6. One reason to give a biological sample for scientific research is to get a medical checkup.^*f*^	2.59 (0.76)	0.748			0.790	0.726
7. One reason to give a biological sample for scientific research is to find out if you have a disease.^*f*^	2.91 (0.67)	0.852			0.826	0.645
8. Information you get by giving a biological sample for scientific research is the best information about your health you could get.^*f*^	2.90 (0.76)	0.820			0.854	0.721

Cells empty if <0.400, SD = standard deviation

^*a*^Benefits: Mean domain score (SD) = 11.40 (2.32); ^*b*^Lay Understanding: Mean domain score (SD) = 5.09 (1.30); ^*c*^Purpose: Mean domain score (SD) = 3.52 (0.97); ^*d*^Pearson product moment; ^*e*^Mean total score (SD) = 20.11 (2.88); ^*f*^Item reverse coded

**Table 3:** Final scale characteristics for one-factor solution

Item	Mean (SD)	Factor loadings	Correlation with total score^*a,b*^
1. A scientific researcher’s number one job is to make sure that the research helps each person who gives a biological sample.^*c*^	2.88 (0.76)	0.647	0.670
2. Researchers mostly do scientific research using biological samples to tell people who give samples if they are sick.^*c*^	2.77 (0.79)	0.773	0.776
3. Researchers will always tell people if their biological sample shows risk for disease.^*c*^	3.01 (0.71)	0.654	0.663
4. One reason to give a biological sample for scientific research is to get a medical checkup.^*c*^	2.59 (0.76)	0.779	0.774
5. One reason to give a biological sample for scientific research is to find out if you have a disease.^*c*^	2.91 (0.67)	0.823	0.806
6. Information you get by giving a biological sample for scientific research is the best information about your health you could get.^*c*^	2.90 (0.76)	0.823	0.811

SD = standard deviation

^*a*^Pearson product moment; ^*b*^Mean total score (SD) = 17.05 (3.35); ^*c*^Item reverse coded

### Instrument validity

Results for the analyses examining construct validity are given in Table 4. For the 3-factor solution, purpose subscores were uncorrelated with genetic knowledge and negatively correlated with researcher trust, while total scores were uncorrelated with research trust, contradicting our *a priori* hypotheses Total scores for the 1-factor solution assessing conflation of biospecimen-based research and clinical care were negatively correlated with genetic knowledge and positively correlated with researcher trust as hypothesized.

**Table 4:** Validation results

	Correlate	Coefficient for 3-factor solution^*a*^	*A priori* expectation	Coefficient for 1-factor solution^*a*^	*A priori* expectation
Lay understanding	GKI	-0.300	Yes		
	Trust	-0.002	Yes		
Purpose	GKI	0.151	No		
	Trust	-0.321	No		
Benefits	GKI	-0.365	Yes		
	Trust	0.330	Yes		
Total Scale	GKI	-0.361	Yes	-0.381	Yes
	Trust	0.147	No	0.381	Yes

GKI = Genetic Knowledge Index; Trust = Hall et al.’s scale measuring trusting medical researchers

^*a*^Pearson product-moment

As the 1-factor solution had superior psychometric properties and evidence of construct validity, we examined differences in the degree of conflation of biospecimen-based research and clinical care by demographic, health care access, and research participation characteristics using *t*-tests and one-way analysis of variance (ANOVA). These results are presented in Table 5. Conflation of research and clinical care differed significantly by employment status, primary language spoken, health insurance type, usual source of health care, and self-rated health. Individuals who were unemployed, spoke only Spanish, had no health insurance, received care at non-traditional venues, and had good self-rated health received higher scores, indicating greater conflation of biospecimen-based research and clinical care. Scores were inversely associated with years of education (test for linear trend, *p*-value < 0.008).

**Table 5:** Univariate analyses of demographic, health care, and research participation variables and conflation of research with biological samples and clinical care

		Mean score (SD)	*p*-value
Overall		17.05 (3.35)	
Age	≤26	16.86 (3.59)	0.752
	27-34	17.53 (3.22)	
	35-49	16.73 (3.88)	
	≥50	17.05 (2.67)	
Gender	Female	16.79 (3.71)	0.341
	Male	17.31 (2.95)	
Employment	Full-time	16.82 (3.25)	<0.002
	Part-time	15.50 (3.21)	
	Unemployed	18.25 (3.17)	
Years education	≤4	18.00 (2.54)	0.054
	5-8	17.77 (3.37)	
	9-12	16.72 (3.50)	
	≥13	15.56 (2.87)	
Annual household income	<$15,000	17.06 (2.85)	0.058
	$15,000 -$34,999	17.31 (3.61)	
	$35,000 -$49,999	16.69 (3.15)	
	>$50,000	12.00 (5.29)	
Primary language spoken	Only Spanish	19.00 (2.91)	<0.001
	Spanish better than English	17.28 (3.02)	
	Both	16.44 (3.28)	
	English better than Spanish	15.60 (3.33)	
	Only English	15.00 (3.16)	
Health insurance	Private	15.43 (3.46)	<0.002
	Government	17.08 (3.23)	
	Both	15.40 (2.70)	
	None	18.51 (2.98)	
Usual source of care	Doctor’s office	15.20 (3.08)	<0.001
	Clinic	17.61 (3.14)	
	Hospital	18.67 (2.80)	
	Other^*a*^	19.0 (4.38)	
Has regular doctor	Yes	16.82 (3.42)	0.153
	No	17.73 (3.06)	
Self-rated health	Excellent or very good	16.08 (4.00)	0.033
	Good	17.69 (3.20)	
	Fair or poor	17.29 (2.05)	
Provided a sample for research in the past	Yes	15.75 (2.49)	0.068
	No	17.11 (3.41)	
	Not sure	19.60 (2.70)	
Would provide a sample for research in the future	Very likely	17.70 (3.49)	0.144
	Somewhat likely	16.75 (3.40)	
	Not likely	16.25 (2.45)	

*p*-values obtained from independent samples *t*-test or one-way analysis of variance using scores from the one factor solution scale

^*a*^Other: Pharmacy, traditional medicine/sobadores, family/friends, or prefer not to answer

## Discussion

We successfully developed a 6-item instrument measuring conflation of observational biospecimen-based research and clinical care for use in Latino communities. The final instrument demonstrated high internal consistency, evidence of content and construct validity, and no evidence of floor and ceiling effects in a convenience sample of 150 Latino adults. It is important to note that the instrument was developed and field-tested in community samples, not exclusively with prior biospecimen donors. Diagnostic misconception can be stringently understood as misconceptions about the likelihood of receiving personal health-related information as a part of research participation in individuals who have provided informed consent and donated a biological sample [2]. Thus, we believe our instrument is best described as assessing conflation of observational biospecimen-based research and clinical care, not diagnostic misconception. Still, this is one of the first quantitative instruments with demonstrated reliability and validity available to measure beliefs related to diagnostic misconception in potential biospecimen donors. Documenting the instrument’s performance characteristics in biospecimen donors, who may differ from potential donors, will allow the instrument to be used at multiple time-points throughout the research process.

Our conceptual model proposed three domains of misconceptions: lay understanding of the distinction between observational biospecimen-based research and clinical trials, understanding of the purpose of biospecimen-based research, and perceived likelihood of personal benefit. This model is similar to the theoretical framework used by Appelbaum et al. to develop their measure of therapeutic misconception, but with unreasonable beliefs about the degree of individualization of the intervention replaced by understanding of the distinction between clinical and observational research [13]. Our analysis did not confirm the three domains proposed in our conceptual model. Two features likely account for the 3-factor solution’s poor psychometric properties. First, many of the items written for the ‘purpose’ and ‘lay understanding’ subscales were dropped due to double loading in the EFA. Thus, these subscales were comprised of only two items and had poor internal consistency [28]. Second, respondents’ tendency to ‘agree’ or ‘strongly agree’ with most items, regardless of whether they reflected accurate or inaccurate perceptions of biospecimen-based research, caused response patterns for these two types of items to vary. That two domains were comprised of all inaccurate items (lay-understanding and benefits), while one was comprised of all accurate items (purpose), also contributed to poor reliability. It is likely that a multidimensional instrument comprised of all accurate or all inaccurate items would have had improved psychometric characteristics. The therapeutic misconception scale, for example, contains all inaccurate statements, despite piloting both accurate and inaccurate items [13]. It is possible that if we had developed and piloted a larger number of items for each domain our ability to distinguish between distinct beliefs related to diagnostic misconceptions would have improved. Alternatively, our conceptual model may be inaccurate or incomplete. Additional theoretical work defining diagnostic misconception and clarifying its manifestation is needed to guide develop of a multidimensional measure.

Latinos are projected to make up 31 % of the US population by 2060 [29], but currently lack robust representation in clinical research funded by the National Institutes of Health as well as large scale biomarker and other in vitro studies that use de-identified biological samples [30, 31]. A growing body of research indicates, however, that Latinos are highly willing to provide biospecimens for research [11, 12, 32, 33]. Eighty four percent of our sample was ‘very’ or ‘somewhat’ likely to provide a sample for research in the future. There is evidence from other populations that individuals may participate in therapeutic and non-therapeutic research as way to monitor their health and access otherwise unavailable health services [34, 35]. Thus, concern that conflation of research participation and clinical evaluation may drive biospecimen donation in medically underserved Latino communities was a primary motivation for this study.

Conflation of biospecimen-based research and clinical care as measured by our instrument did not differ by self-reported willingness to participate in biospecimen research in our sample (*p* = 0.144). Still, we found that Latino subgroups facing the most substantial barriers to accessing high quality health care had higher scores, indicating a greater degree of conflation. Those who were unemployed, spoke only Spanish, had no health insurance, and received health care outside of traditional venues were more likely to conflate aspects of research and clinical care. These groups stand to face a disproportionate burden of the potential harms resulting from diagnostic misconception, which may include damaged trust in both doctors and researchers [5].

Efforts to recruit Latinos into biospecimen-based research must avoid paternalism, but also recognizing that biospecimen donation is not without risk. Additional research is needed to determine whether Latinos’ with limited access to traditional health care experience undue influence in the research setting and the role that diagnostic misconception plays in this process. While mean scores did not differ between those who reported prior biospecimen donation in a one-way ANOVA, individuals who reported providing a sample for research in the past tended to have lower scores than those who had not (15.75 vs. 17.11) and a corresponding non-parametric test was significant (Kruskal-Wallis *p*-value < 0.031). Thus, it is possible the recruitment and informed consent process may help clarify misconceptions about the purpose and benefits of observational research compared to clinical care. Alternatively, those with access to research participation opportunities may be more knowledgeable about research. These are questions that should be assessed in future longitudinal studies with biospecimen donors.

This study had several limitations. We surveyed Mexican-Americans living in a rural, agricultural community using non-probability sampling, limiting the generalizability of our findings to other Hispanic and Latino communities in the US. A recent focus group study with Puerto Ricans living in Buffalo, New York with similar income, education, and health care access characteristics to our participants reported that “the inability to conceptualize the difference between biomedical research and medical diagnostic services and results” was common ([32], p465). This suggests that our results may have broader applicability across Latino subpopulations. The instruments used for validation had poor internal consistency in our population, which could have affected our results. Additionally, we did not assess item ordering effects, reproducibility, or responsiveness of the survey instrument. Our instrument can be easily implemented in other settings to examine these properties. Because a gold standard does not exist, we could not assess criterion validity. Also, because we did not include a ‘do not know’ option we are unable to tell if participants misunderstood the question, did now know enough about research to make an educated guess, or truly had misperceptions about research with biological samples. Results from a recent effort to develop a biobanking knowledge scale in South Florida suggest that almost half of respondents respond ‘do not know’ to knowledge items [36]. Finally, we did not establish the interpretability of our instrument by assigning qualitative meaning to quantitative scores. Understanding the degree of conflation that could compromises participation decision-making and establishing cut-off scores identifying groups at risk of experiencing diagnostic misconception during the recruitment and informed consent process will be an important goal for future studies.

## Conclusions

The availability of a quantitative instrument measuring conflation of research with biological samples and clinical care will enable researchers to better flesh out how Latinos make decisions to participate in biospecimen-based research in a context of health care inequality. The final 6-item survey instrument can be used to assess baseline knowledge and beliefs about biospecimen-based research to guide community engagement activities prior to recruitment or to evaluate educational interventions or the quality of the informed consent process. Efforts to improve public health through large-scale biospecimen-based studies are predicated on investigators’ ability to engage diverse populations. It is imperative that attempts to increase Latinos’ representation in population-based biobanking research do not inadvertently exploit this community’s limited access to clinical services and desire for health information [37]. A better understanding of diagnostic misconception will help ensure that decisions to donate biospecimens are made based on an informed weighing of the current risks and benefits of research participation.

## Additional file

Additional file 1:
**Supplementary Table S1-S3 reporting item-level data for all piloted items.**

## Abbreviations

US: United States
NCI: National Cancer Institute
GKI: Genetic Knowledge Index
EFA: Exploratory Factor Analysis
CNPC: Community Networks Program Center
ANOVA: Analysis of Variance
