# Supplementary material for: Giving samples or “getting checked”: measuring conflation of observational biospecimen research and clinical care in Latino communities
Source: BMC Med Ethics. 2015 Jul 17;16:49. doi: 10.1186/s12910-015-0041-9 (PMC4504441; doi:10.1186/s12910-015-0041-9)
Supplement: Additional file 1: — Supplementary Table S1-S3 reporting item-level data for all piloted items. [file 12910_2015_41_MOESM1_ESM.pdf]

Supplementary Table 1. Item-level data for domain one of piloted survey instrument.

Domain	Item	Frequency						Mean	SD
			1	2	3	4	Missing		
Lay understanding	1. A person must be sick to give a sample for scientific research.[a]	n	21	89	32	8	0	2.18	0.73
		%	14.0	59.3	21.3	5.3	0		
	2. A person is not required to take medicine when giving a biological sample for scientific research.	n	19	64	57	10	0	2.39	0.79
		%	12.7	42.7	38.0	6.7	0		
	3. A person must go to the hospital to give a biological sample for scientific research.[a]	n	9	48	73	20	0	2.69	0.78
		%	6.0	32.0	48.7	13.3	0		
	4. A healthy person can give a biological sample for scientific research.	n	50	92	7	1	0	1.73	0.58
		%	33.3	61.3	4.7	0.7	0		
	5. A person must be invited by a doctor to give a biological sample for scientific research.[a]	n	13	63	58	45	1	2.50	0.79
		%	8.7	42.0	38.7	10.0	0.7		
	6. Scientific research using biological samples is mainly done to test if new medical treatments are safe for humans.[a,b]	n	4	16	95	33	2	3.06	0.66
		%	2.7	10.7	63.3	22.0	1.3		

Note: SD=standard deviation; Response categories: 1= strongly agree; 2=agree; 3=disagree; 4=strongly disagree.

[a]Items were reverse coded, so that a higher score indicates presence of a misconception; [b]Skewed item.

Supplementary Table 2. Item-level data for domain two of piloted survey instrument.

Domain	Item	Frequency						Mean	SD
			1	2	3	4	Missing		
Purpose	1. The purpose of scientific research using biological samples is to give the best medical information to those who give samples.[a]	n	0	20	99	31	0	3.07	0.58
		%	0	13.3	66.0	20.7	0		
	2. Scientific research using biological samples is done to help the people who give samples, not to help future generations.[a]	n	20	58	57	15	0	2.45	0.85
		%	13.3	38.7	38.0	10.0	0		
	3. A scientific researcher's number one job is to make sure that the research helps each person who gives a biological sample.[a]	n	6	35	80	29	0	2.88	0.76
		%	4.0	23.3	53.3	19.3	0		
	4. Researchers mostly do scientific research using biological samples to tell people who give samples if they are sick.[a]	n	7	47	70	26	0	2.77	0.79
		%	4.7	31.3	46.7	17.3	0		
	5. Scientific research using biological samples is done to learn about what causes disease, not about each person who gives a biological sample.	n	38	93	17	2	0	1.89	0.64
		%	25.3	62.0	11.3	1.3	0		

	6. The goal of scientific research with biological samples is help people in the future, whether or not it helps each person who gives a sample.[b]	n	50	89	9	2	0	1.75	0.62
		%	33.3	59.3	6.0	1.3	0		
	7. A scientific researcher's number one goal is to learn more about how to fight disease.	n	59	86	4	0	1	1.63	0.54
		%	39.3	57.3	2.7	0	0.7		

Note: SD=standard deviation; Response categories: 1= strongly agree; 2=agree; 3=disagree; 4=strongly disagree.

[a]Items were reverse coded, so that a higher score indicates presence of a misconception; [b]Skewed item.

Supplementary Table 3. Item-level data for domain three of piloted survey instrument.

Domain	Item	Frequency						Mean	SD
			1	2	3	4	Missing		
Benefits	1. Researchers will always tell people if their biological sample shows risk for disease.[a]	n	4	25	87	34	0	3.01	0.71
		%	2.7	16.7	58.0	22.7	0		
	2. Giving a biological sample for scientific research may not give you any important information about your health.	n	19	69	54	8	0	2.34	0.77
		%	12.7	46.0	36.0	5.3	0		
	3. One reason to give a biological sample for scientific research is to get a medical checkup.[a]	n	11	54	71	14	0	2.59	0.76
		%	7.3	36.0	47.3	9.3	0		
	4. By giving a biological sample for scientific research, a person will always get important information about their disease risk.[a]	n	3	28	93	26	0	2.94	0.66
		%	2.0	18.7	62.0	17.3	0		
	5. When you give a biological sample for scientific research you always learn if you might get the disease they are studying.[a]	n	2	36	92	20	0	2.87	0.64
		%	1.3	24.0	61.3	13.3	0		
	6. One reason to give a biological sample for scientific research is to find out if you have a disease.[a]	n	5	26	96	23	0	2.91	0.67
		%	3.3	17.3	64.0	15.3	0		

	7. Information you get by giving a biological sample for scientific research is the best information about your health you could get.[a]	n	5	36	77	31	1	2.90	0.76
		%	3.3	24.0	51.3	20.7	0.7		

Note: SD=standard deviation; Response categories: 1= strongly agree; 2=agree; 3=disagree; 4=strongly disagree.

[a]Items were reverse coded, so that a higher score indicates presence of a misconception.
